# Magnitude Estimates Orchestrate Hierarchal Construction of Context-Dependent Representational Maps for Vestibular Space and Time: Theoretical Implications for Functional Dizziness

**DOI:** 10.3389/fnint.2021.806940

**Published:** 2022-02-04

**Authors:** Qadeer Arshad, Yougan Saman, Mishaal Sharif, Diego Kaski, Jeffrey P. Staab

**Affiliations:** ^1^Neuro-Otology Unit, Department of Brain Sciences, Charing Cross Hospital Campus, Imperial College London, London, United Kingdom; ^2^inAmind Laboratory, Department of Neuroscience, Psychology and Behaviour, University of Leicester, Leicester, United Kingdom; ^3^Department of Clinical and Motor Neurosciences, Institute of Neurology, University College London, London, United Kingdom; ^4^Departments of Psychiatry and Psychology and Otorhinolaryngology – Head and Neck Surgery, Mayo Clinic, Rochester, MN, United States

**Keywords:** vestibular cortex, functional dizziness, space perception, time perception, interhemisphere asymmetry

## Abstract

Maintaining balance necessitates an accurate perceptual map of the external world. Neuro-physiological mechanisms of locomotor control, sensory perception, and anxiety systems have been viewed as separate entities that can on occasion affect each other (i.e., walking on ice). Emerging models are more integrated, that envision sensory perception and threat assessment as a fundamental component of balance. Here we present an empirically based theoretical argument that vestibular cortical areas construct magnitude estimates of our environment *via* neural integration of incoming sensory signals. In turn, these cortically derived magnitude estimates, construct context-dependent vestibulo-spatial and vestibulo-temporal, representational maps of the external world, and ensure an appropriate online scaling factor for associated action-perceptual risk. Thus, threat signals are able to exert continuous influence on planning movements, predicting outcomes of motion of self and surrounding objects, and adjusting tolerances for discrepancies between predicted and actual estimates. Such a process affects the degree of conscious attention directed to spatial and temporal aspects of motion stimuli, implying that maintaining balance may follow a Bayesian approach in which the relative weighting of vestibulo-spatial and vestibulo-temporal signals and tolerance for discrepancies are adjusted in accordance with the level of threat assessment. Here, we seek to mechanistically explain this process with our novel empirical concept of a Brainstem Cortical Scaling Metric (BCSM), which we developed from a series of neurophysiological studies illustrating the central role of interhemispheric vestibulo-cortical asymmetries for balance control. We conclude by using the BCSM to derive theoretical predictions of how a dysfunctional BCSM can mechanistically account for functional dizziness.

## Overview

Patients with psychosomatic vestibular conditions (i.e., functional dizziness) have been extensively studied over the last four decades. Various terms have emerged to describe these patients including phobic postural vertigo (Brandt, 1996), space-motion discomfort (Jacob et al., 1993), visual-vertigo (Bronstein, 1995), and chronic subjective dizziness (Ruckenstein and Staab, 2009). A new umbrella term has emerged to describe patients with functional dizziness, namely, Persistent Postural Perceptual Dizziness (PPPD) (Staab et al., 2018). The definition of PPPD was promulgated by an international panel of experts convened by the Bárány Society and is included in the 11th edition of the International Classification of Diseases (ICD-11; WHO, 2015a,b; Staab et al., 2018).

Clinically, patients with PPPD present with two key fluctuating or continuous symptoms; (a) a dizzy, not-truly vertiginous sensation, with patients reporting that their head is swimming and/or (b) unsteadiness, such that patients report swaying, rocking, or jelly legs. Symptoms can be exacerbated in visually complex environments, during upright posture and head movements. Typically, PPPD is triggered by an acute disruption to normal balance function; however, the development of PPPD is not attributable to the degree of otological dysfunction or failed ear recovery. Rather, psychological risk factors (anxiety-related personality traits) and shifts in psycho-physical functioning (space-motion perceptual style and visual dependence) are strong predictive factors for determining which patients will develop PPPD following vestibular dysfunction (Cousins et al., 2017). This is in line with clinical histories that illustrate symptom severity is modulated by factors such as introspection, distraction, fatigue, and alertness.

**FIGURE 1:**
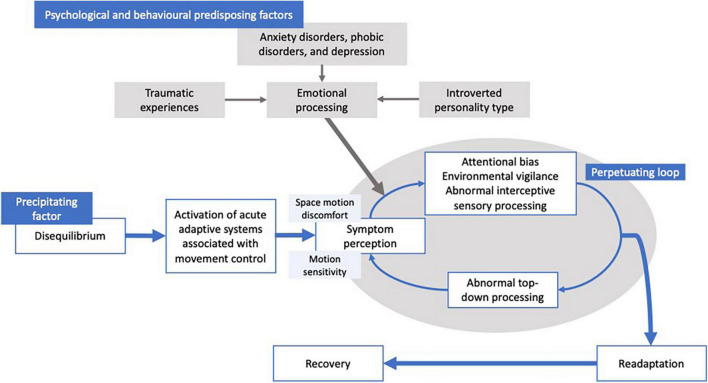
Graphical depiction of the mechanisms suggested to be involved in functional dizziness. That is, a participating factor (i.e., vestibular loss, medical cause, and anxiety) causes disequilibrium, leading to a transient distortion of vestibular perception impairing cognitive control of movement and balance. Psychological factors and behavioral co-morbidities (i.e., anxiety, phobia, emotional trauma, and personality types) are suggested to increase the likelihood of the transient perceptual distortions to persist, resulting in a perpetuating loop causing a failure of re-adaptation.

Such a clinical picture gives rise to the notion that functional dizziness is in essence a perceptual disorder, as illustrated by the schematic (Figure 1) of the putative pathophysiological mechanisms implicated for functional dizziness. Indeed, our vestibular signals are critical for facilitating the detection of body motion *via* spatial and temporal perceptual mechanisms. Elucidating the neurobiological basis of functional dizziness is clinically pertinent, especially when considering its high prevalence (20% of dizzy patients in general neurology clinics; 40% in specialized dizziness centers; 5% of general population), as well as the considerable functional (80% limiting daily social activities), occupational (41% take time off work), and cognitive impairment imparted (Dieterich et al., 2016; Xue et al., 2018; Staibano et al., 2019; Adamec et al., 2020; Kim et al., 2020).

Despite emerging evidence, which we shall review here, exactly how the brain derives vestibulo-spatial and vestibulo-temporal perceptual maps of the external world remains unclear. Furthermore, whether distorted representational maps of space and time can account for symptoms in functional patients that exhibit disordered vestibular perception, remains unclear. To address these questions, in this review, we shall consider emerging research regarding how vestibular-related brain mechanisms code for space and time and their associated implications for movement and balance in healthy individuals. Consequently, this will enable us to make theoretically derived predictions of how balance and movement are impaired in the pathophysiological state.

## Proposition

We propose that the pathophysiological mechanisms underlying PPPD involve alterations in processing of multi-modal (i.e., vestibular, visual, proprioceptive, auditory) motion stimuli (brainstem to cortical pathways), changes in conscious awareness of motion, and reduced tolerance for both perceived postural instability (intra-cortical networks) and resulting adjustments to locomotor control strategies (cortical to brainstem and spinal cord pathways). These bottom-up and top-down alterations in functioning are affected by over-vigilant threat assessments (i.e., high trait anxiety and excessive body vigilance) which in turn induce greater physiological and psychological reactivity to discrepancies between actual and perceived motion (Figure 1). Based on such a model, one would predict that patients with functional dizziness will exhibit, (a) altered interhemispheric brainstem-cortical functional connectivity, (b) perceptually biased estimates of space and displacement, and (c) erroneous predictions of self-motion perception. Furthermore, abnormalities (a–c) would be correlated with psychological measures such as trait anxiety to ensure the scaling factor is maintained and proportionate. Indeed, such a proposition is broadly consistent with other models of functional neurological disorders, that posit abnormal integration between top-down and bottom-up brain systems that monitor and regulate behavior (Edwards and Bhatia, 2012; Edwards et al., 2012; Stone and Edwards, 2012).

## Magnitude–Space–Time

Testing the proposition of abnormal integration between brainstem and cortical processes is challenging owing to gaps that exist in vestibular neuroscience regarding how these processes principally interact. Brainstem mechanisms are posited to construct magnitude estimates of self-displacement by mathematically integrating angular and linear head velocity signals *via* a process that can be viewed as an extension of “velocity storage” and “neural integration” (Cohen et al., 1981). These bottom-up vestibular transformations and their subsequent sensory integration feed into the vestibulo-cortical network to maintain spatial orientation. Thus, the vestibular cortex must resolve spatial and temporal ambiguity that requires the ability to formulate an internalized magnitude estimate and/or judgment, for example, “am I moving or are objects moving around me,” and “how long have I been moving”? Consideration of these spatial- and temporal-based questions highlights a key commonality across the dimension of space and time that the brain must extract and utilize in order to resolve perceptual ambiguity, which we propose is magnitude perception (Walsh, 2003).

Our emerging data implicate the vestibular cortex in setting the parameters of the dimensional entities of representational magnitude, space, and time in a set hierarchal pattern, with dynamic interhemispheric competition representing the control mechanism. This is supported by our data that illustrates numerical magnitude perception (i.e., estimating the midpoint between two intervals; 23–87) can be selectively biased toward smaller or larger magnitudes in a hemispheric dependent fashion, and that such biasing can disrupt the construction of representational space and time (Arshad et al., 2016; Kaski et al., 2016). Central to this proposition is that vestibulo-cortical control, magnitude allocation, and spatial attention are subject to dynamic interhemispheric competition, implying a common cortical control mechanism (Kinsbourne, 1977; Dieterich et al., 2003; Arshad et al., 2016). Indeed, data from patients with cortical lesions, which disrupt normal interhemispheric interactions, have illustrated the presence of cortical and brainstem mediated biases in both the vestibulo-spatial and vestibulo-temporal domains (Rubens, 1985; Ventre-Dominey et al., 2003). Of note, the functional relevance of interhemispheric interactions is provided by data that demonstrate that non-invasive modulation of the healthy hemisphere in stroke patients can ameliorate the pathological biases following stroke (Sparing et al., 2009). Functionally speaking, such cortical co-arrangement facilitates the integration of information across the physical dimensions of magnitude, space, and time, allowing for the utilization of expected vestibular signals to construct the intended trajectory of self-locomotion compared to vestibular feedback indicating the accuracy of actual movement. To provide empirical data for this proposition, we have developed novel neurophysiological techniques to disrupt interhemispheric interactions in healthy individuals and adapted experimental paradigms for use in patients with vestibular lesions.

## Experimental Techniques to Probe the Brainstem-Cortical Scaling Metric

**FIGURE 2:**
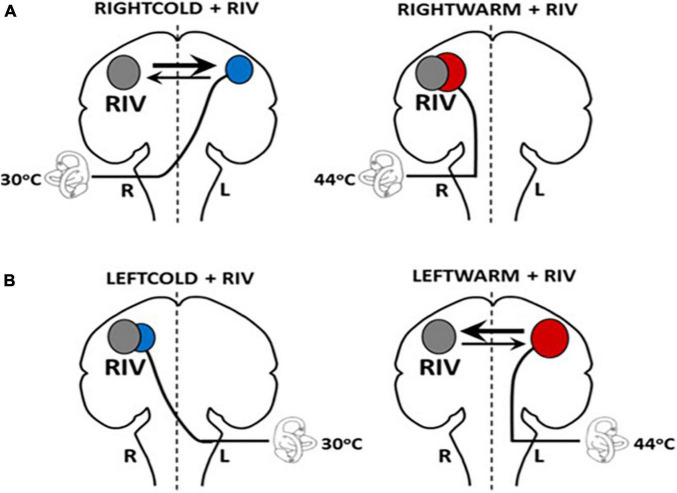
Schematic model illustrating hemispheric activation during CALORIC + RIV stimulation: perceptual switching during binocular rivalry activates the right hemisphere (gray circle). Hemispheric activations following caloric stimulation are shown by the red circle following warm irrigations or by blue circles following cold irrigations. The labyrinth represents the side of the caloric irrigation. The size of the circles illustrates the relative degree of the activation. **(A)** During RIGHTCOLD + RIV, the hemispheres are in conflict; however, the right hemisphere exerts a predominant effect (as shown by the relative thickness of the arrows). No interhemispheric conflict occurs during either RIGHTWARM + RIV as both stimuli activate the right hemisphere. **(B)** Similarly, no conflict is present in LEFTCOLD + RIV condition, whereas during the LEFTWARM + RIV condition, conflict presents, but critically here the left hemisphere exerts a greater influence.

One of the techniques we developed to disrupt interhemispheric interactions involves combining binocular rivalry (RIV) (different images simultaneously presented to each eye which cannot be fused resulting in the eyes competing for perceptual dominance) with vestibular stimulation (i.e., caloric irrigations with either cold (30°C) or warm (44°C) water irrigations), referred to as CAL+RIV stimulation. Notably, this paradigm can selectively induce interhemispheric competition when rivalry is combined with caloric irrigations that preferentially implicate the left hemisphere [i.e., right-ear cold (RIGHTCOLD + RIV) and left-ear warm (LEFTWARM + RIV)] (Figure 2). No conflict or biases occur when caloric irrigations preferentially implicate the same (i.e., right) hemisphere as the rivalry stimulus [i.e., left-ear cold irrigations (LEFTCOLD + RIV) or right-ear warm irrigations (RIGHTWARM + RIV)]. Indeed, experimental application of this technique reveals an ability, during the conflict conditions, to bias cognitive processes and vestibular processing [namely suppression of the brainstem mediated vestibular-ocular reflex (VOR)] in a manner consistent with that expected following unihemispheric inhibition (see the section below for further details) (Arshad et al., 2016).

**FIGURE 3:**
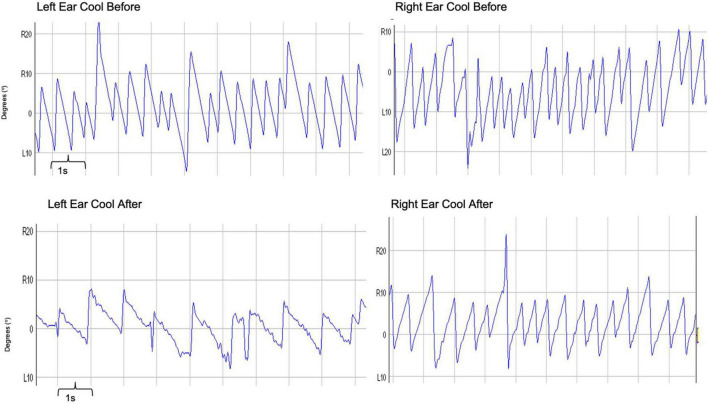
Trace depicting caloric-induced vestibular nystagmus: Raw traces obtained before and after the application of left hemisphere cathodal tDCS. Right-ear cold irrigations are represented in the right panel, whereas left-ear cold irrigations in the left panel. The recordings before tDCS are on the top with after tDCS traces represented on the bottom. The *x*-axis represents time, with one large square representing 1 s. The *y*-axis represents the degrees of eye movement either right (R) or left (L) from the center (0°). To measure the slow phase velocity of a nystagmus, one utilizes the slope of the nystagmus in its slow phase. In these recordings, one can clearly see a less steep slope, and therefore a suppressed slow phase velocity of the nystagmus following cathodal tDCS – for both right and left ear cold irrigations.

The second technique involves modulating vestibulo-cortical excitability over the posterior parietal cortex (PPC), with transcranial direct current stimulation (tDCS). Application of unipolar cathodal tDCS over the left PPC induces top-down modulation of the VOR. Right hemisphere cathodal stimulation does not modulate the VOR, despite housing the predominant vestibular representation. This lack of modulation following (right hemisphere) cathodal stimulation can be accounted for by an on-going functional interhemispheric asymmetry (i.e., in right handers, for spatial/vestibular functions the right hemisphere is more dominant) between the parietal cortices enabling the right hemisphere to exert greater inhibition over the left, in right-handed individuals. Accordingly, cathodal tDCS of the left PPC not only inhibits its ability to process vestibular signals but also potentiates right hemispheric dominance (Figure 3; Arshad et al., 2014).

Both techniques reviewed above, namely CAL + RIV stimulation and tDCS, suppress the VOR *via* top-down vestibulo-cortical mechanisms. Specifically, the suppression in the amplitude of elicited vestibular nystagmus reflects the degree of interhemispheric modulation using either CAL + RIV stimulation or tDCS, and thus reflects the brainstem contribution to the proposed Brainstem Cortical Scaling Metric (BCSM). Quantification of this brainstem component is made by calculating the nystagmus suppression index (NSI) which is the percentage change (before vs. after CAL + RIV or tDCS) in the peak slow phase velocity of the VOR (Arshad et al., 2015).

## Significance of the Brainstem-Cortical Scaling Metric on Magnitude, Space, and Time Perception

**FIGURE 4:**
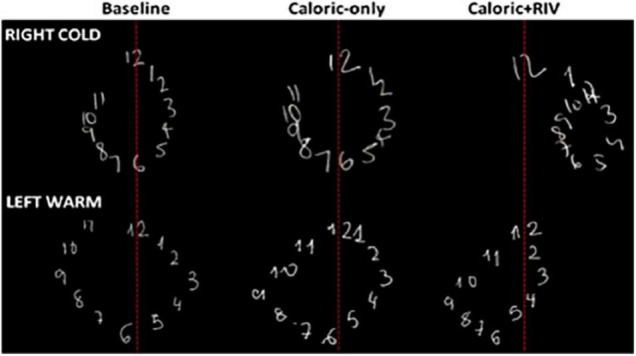
Representative numerical clock drawings from a single subject during CALORIC + RIV stimulation: upper panel shows the drawings for the baseline condition (darkness), following RIGHTCOLD caloric only and RIGHTCOLD + RIV. Note the rightward lateral displacement of the numerical clock drawing following RIGHTCOLD + RIV. The lower panel shows the drawings for the baseline condition (darkness), following LEFTWARM caloric only and LEFTWARM + RIV. Note the leftward lateral displacement of the numerical clock drawing following LEFTWARM + RIV.

Using the techniques reviewed above to disrupt interhemispheric interactions not only disrupts low-level vestibular function (i.e., suppression of brainstem mediated VOR) but can additionally bias magnitude, spatial, and temporal percepts in a proportional manner (Arshad et al., 2015, 2016; Nigmatullina et al., 2016). That is, biased percepts are correlated with the extent of the VOR suppression (i.e., NSI), ensuring modulated brainstem estimates of head displacement match perceptual (“cortically based”) estimates. For example, RIGHTCOLD + RIV stimulation when compared to the corresponding caloric alone condition (RIGHTCOLD) biases numerical magnitude estimates (mid-point between two numbers) toward smaller magnitudes, mediated by a right-hemisphere predominant response following interhemispheric conflict, as corroborated by lesion data (Zorzi et al., 2002). LEFTWARM + RIV stimulation, when compared to the corresponding caloric alone condition (LEFTWARM), biases magnitude estimates toward larger magnitudes, mediated by a left-hemisphere predominant response (Arshad et al., 2016). To explore the influence of these magnitude biases upon representational space, we asked subjects to draw numerical (magnitude-dependent, 1–12) or alphabetical (magnitude-independent, A–L) clock faces with their eyes closed. Lateral distortions were observed only for the numerical, but not the alphabetical clock drawings. Critically, these occurred in the opposite direction to that predicted by a hemispheric-mediated spatial biasing account, implying that magnitude supersedes the representational construct of space (Figure 4; Arshad et al., 2016).

**FIGURE 5:**
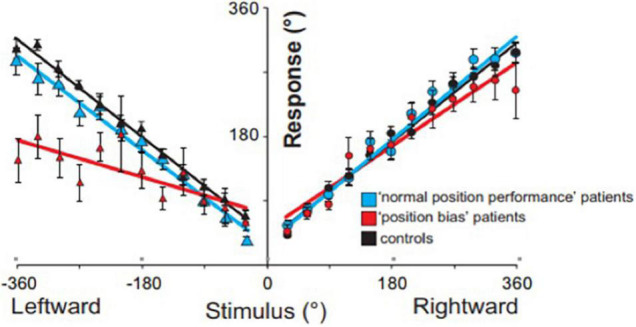
Position task results: grouped response–stimulus position performances are shown for patients with a position bias (red; ‘patients), patients with normal performance (blue; control patients), and age-matched controls (black line). Position bias was calculated for each stroke patient from the subjective response–stimulus position performance regressions, by dividing the leftward regression slope by the rightward regression slope. Vertical bars represent standard errors of the mean.

Regarding temporal biasing, right hemispheric lesions bias magnitude percepts toward larger magnitude (Zorzi et al., 2002). Based on the above findings, we predicted that if right-hemispheric lesion patients were asked to imagine a clock face, it would disrupt temporal estimates of self-displacement inside an imagined clock face due to an associated expansion of leftward representational space. As shown in Figure 5, stroke patients underestimated their perceived displacement during leftward rotations despite having normal motion perception, due to the fact that they felt as though they traveled less distance in the same time (i.e., expanded space) (Kaski et al., 2016).

Taken together, our findings imply that disrupting interhemispheric interactions alters vestibulo-cortical functioning in turn biasing magnitude percepts that subsequently distorts the coding of representational space and time. Indeed, we have proceeded to demonstrate that such distortion of space and time impairs control of body motion as illustrated by our recent findings in healthy individuals.

## Relevance and Predictions for Functional Balance and Movement Disorders

**FIGURE 6:**
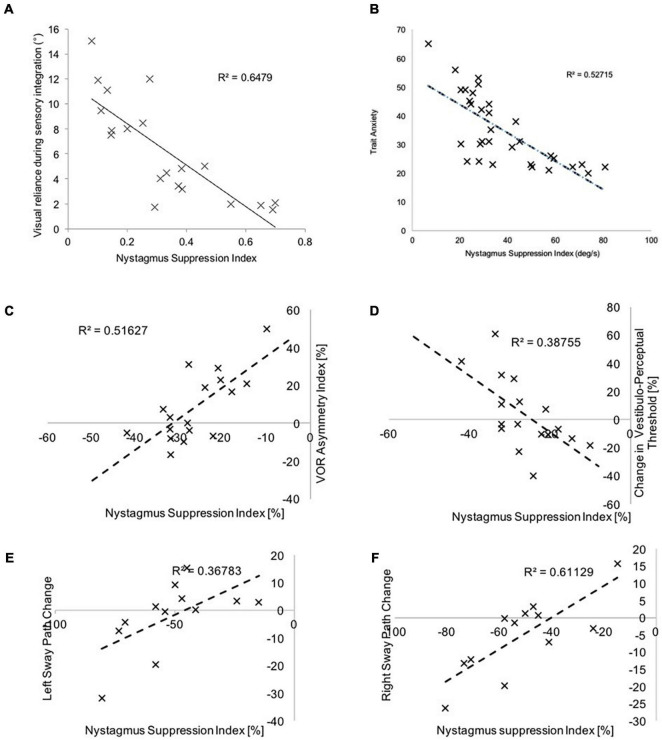
Here we represent the relationship between the NSI (following left PPC cathodal stimulation) and **(A)** visual dependency-assessed using the rod and disk task, **(B)** trait anxiety assessed using the Spielberger questionnaire, **(C)** vestibulo-ocular thresholds during yaw rotations, **(D)** vestibulo-perceptual thresholds during yaw rotations, **(E)** sway velocity during leftward body motion and, **(F)** sway velocity during rightward motion. Greater NSI indicates increased right-hemispheric vestibulo-cortical dominance, and this is associated with **(A)** reduced reliance upon visual cues during sensory integration, **(B)** reduced trait anxiety, **(C,D)** reduced motion detection thresholds, and, **(D,E)** reduced sway velocity.

Our data in healthy individuals (Figure 6), illustrates that following cathodal stimulation of the left PPC, increased right hemispheric vestibulo-cortical dominance (i.e., a good vestibular inertial system as reflected by greater NSI) is associated with (1) reduced visual dependency (Arshad et al., 2019), (2) lower trait anxiety (Bednarczuk et al., 2018), (3) reduced motion sensitivity (Arshad et al., 2019), and (4) increased postural stability (Castro et al., 2019). These factors are suggested to reduce the risk factors for developing functional dizziness. Thus, we postulate that in right-handers, increased right hemispheric vestibulo-cortical dominance is likely to protect against the development of functional dizziness.

Accordingly, we postulate that patients with functional dizziness will exhibit reduced right-lateralized vestibulo-cortical dominance manifesting distorted representations of space and time attributable to biased percepts toward larger magnitudes. In turn, the BCSM’s ability to properly scale estimates of displacement and duration is altered, thereby throwing off internal predictions of space and time, which in turn impairs accurate assessment of errors between predicted and actual motion, resulting in sensations of disconnectedness from self and surroundings (mild depersonalization and derealization). These minor dissociative symptoms are consistently experienced by patients with chronic dizziness, including functional dizziness, and by normal individuals exposed to strong vestibular stimuli (Jáuregui-Renaud et al., 2008; Aranda-Moreno and Jáuregui-Renaud, 2016). Such sensations of foggy headedness or indistinct feelings of not being well planted in space disrupt normal reflexive processes. That is, under normal circumstances, humans are largely unaware of the details of sensory integration, motion perception, and locomotor control, which operate outside of executive processes (fully conscious perception and action). These sensorimotor functions may transiently intrude into consciousness as when traversing an icy pavement, but even then, most aspects of locomotor dynamics (e.g., individual muscle movements) remain outside of direct executive control. Patients with functional dizziness, however, experience a consistently heightened, conscious awareness of space and motion stimuli, particularly an increased sense of error between predicted (conscious) and actual (subconscious) motions. Driven by their anxious temperaments, functional dizzy patients exert unnecessarily increased executive control over locomotion, altering reflexive postural dynamics and normal weighting of sensory inputs, a mismatched behavioral response that paradoxically reduces the effectiveness of lower-level systems. Research on heuristics in decision-making has well described the adverse effects of undue emphasis on risk and the influences of emotional states induced by perceptual biases (Kahneman, 2003).

## Theoretically Derived Prediction

**FIGURE 7:**
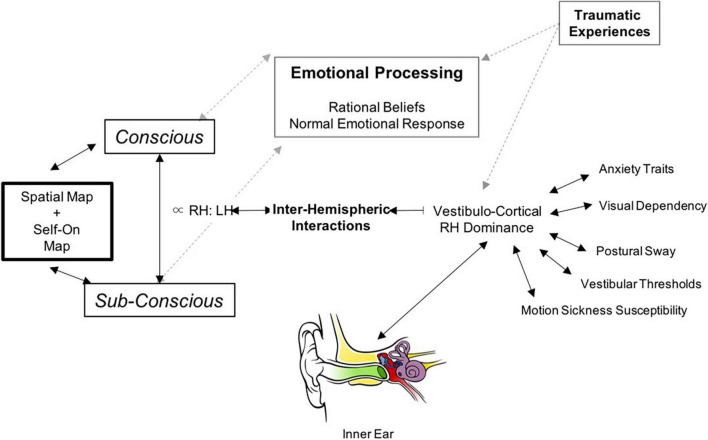
Schematic illustration of our proposed neurobiological model for PPPD: The central component in the model is the interhemispheric interactions in the vestibulo-cortical network that determines its dominance and in-turn influences risk factors for developing PPPD including lower motion perceptual thresholds, increased sway during postural control, increased motion sickness susceptibility, higher visual dependence, and anxiety. These interhemispheric interactions can also be influenced by predisposing emotional trauma (non-vestibular). This cortical network receives signals from bottom-up vestibular transformations provided by the vestibular system. The net result of the cortical system is to generate perceptual vestibulo-spatial and vestibulo-temporal maps of the world, and this is cross-referenced with physical signals from the accelerometers (balance organ) in our inner ear. If there is a mismatch as in the case of PPPD – this subsequently opens up feed-forward pathways to emotional networks.

The outlined conceptual principles above require a pathophysiological model of functional dizziness that can tie together threat assessment, risk tolerance, perceptual bias, and functional shifts in space-motion processing and locomotor control. Accordingly, we propose the following theoretical hypothesis and schematic model as depicted in Figure 7. Namely, in patients with PPPD, reduced right hemispheric vestibulo-cortical dominance, (1) alters cortical processing of brainstem mediated head velocity signals, thereby distorting interhemispheric representations of the magnitudes–space–time continuum, biasing dynamic perceptual maps of body position and motion in the physical world and (2) concurrently heightens threat assessment and reduced risk tolerance from an anxious temperament. In turn, this disrupts the normal process of detecting errors between predicted (conscious) and actual (subconscious) movement, which intrusively and unnecessarily rises to the level of conscious awareness and impacts upon emotional networks. Such multi-level changes in brain functioning account for the primary symptoms of functional dizziness that patients experience, including a diminished sense of agency about control of locomotion. Future research and experimental data will determine the validity of the proposed theoretical predictions made herewith.

## Author Contributions

QA conceptualized and wrote the manuscript. YS provided the technical contributions and edited the manuscript. MS edited the manuscript and drafted the figures. DK and JS conceptualized and edited the manuscript. All authors contributed to the article and approved the submitted version.

## Conflict of Interest

The authors declare that the research was conducted in the absence of any commercial or financial relationships that could be construed as a potential conflict of interest.

## Publisher’s Note

All claims expressed in this article are solely those of the authors and do not necessarily represent those of their affiliated organizations, or those of the publisher, the editors and the reviewers. Any product that may be evaluated in this article, or claim that may be made by its manufacturer, is not guaranteed or endorsed by the publisher.

## References

[B1] Adamec I. Meaški S. J. Skorić M. K. Jažić K. Crnošija L. Milivojević I. (2020). Persistent postural-perceptual dizziness: clinical and neurophysiological study. *J. Clin. Neurosci.* 72 26–30. 10.1016/j.jocn.2020.01.043 31948878

[B2] Aranda-Moreno C. Jáuregui-Renaud K. (2016). Derealization during utricular stimulation. *J. Vestib. Res.* 26 425–431. 10.3233/VES-160597 28262646

[B3] Arshad Q. Nigmatullina Y. Nigmatullin R. Asavarut P. Goga U. Khan S. (2016). Bidirectional modulation of numerical magnitude. *Cereb. Cortex* 26 2311–2324. 10.1093/cercor/bhv344 26879093 PMC4830300

[B4] Arshad Q. Nigmatullina Y. Roberts R. E. Bhrugubanda V. Asavarut P. Bronstein A. M. (2014). Left cathodal trans-cranial direct current stimulation of the parietal cortex leads to an asymmetrical modulation of the vestibular-ocular reflex. *Brain Stimul.* 7 85–91. 10.1016/j.brs.2013.07.002 23941985 PMC3893485

[B5] Arshad Q. Ortega M. C. Goga U. Lobo R. Siddiqui S. Mediratta S. (2019). Interhemispheric control of sensory cue integration and self-motion perception. *Neuroscience* 408 378–387. 10.1016/j.neuroscience.2019.04.027 31026563

[B6] Arshad Q. Siddiqui S. Ramachandran S. Goga U. Bonsu A. Patel M. (2015). Right hemisphere dominance directly predicts both baseline V1 cortical excitability and the degree of top-down modulation exerted over low-level brain structures. *Neuroscience* 311 484–489. 10.1016/j.neuroscience.2015.10.045 26518461 PMC4674775

[B7] Bednarczuk N. F. Casanovas Ortega M. Fluri A. Arshad Q. (2018). Vestibulo-cortical hemispheric dominance: the link between anxiety and the vestibular system? *Eur. J. Neurosci.* 47 1517–1524. 10.1111/ejn.13948 29768682 PMC6099323

[B8] Brandt T. (1996). Phobic postural vertigo. *Neurology* 46 1515–1519. 10.1212/wnl.46.6.1515 8649539

[B9] Bronstein A. M. (1995). The visual vertigo syndrome. *Acta Otolaryngol.* 115 45–48. 10.3109/00016489509125186 8749077

[B10] Castro P. Kaski D. Al-Fazly H. Ak D. Oktay L. Bronstein A. (2019). Body sway during postural perturbations is mediated by the degree of vestibulo-cortical dominance. *Brain Stimul.* 12 1098–1100. 10.1016/j.brs.2019.05.008 31105028

[B11] Cohen B. Henn V. Raphan T. Dennett D. (1981). Velocity storage, nystagmus, and visual-vestibular interactions in humans*. *Ann. N. Y. Acad. Sci.* 374 421–433. 10.1111/j.1749-6632.1981.tb30888.x 6978639

[B12] Cousins S. Kaski D. Cutfield N. Arshad Q. Ahmad H. Gresty M. A. (2017). Predictors of clinical recovery from vestibular neuritis: a prospective study. *Annal. Clin. Transl. Neurol.* 4 340–346. 10.1002/acn3.386 28491901 PMC5420806

[B13] Dieterich M. Bense S. Lutz S. Drzezga A. Stephan T. Bartenstein P. (2003). Dominance for vestibular cortical function in the non-dominant hemisphere. *Cereb. Cortex* 13 994–1007. 10.1093/cercor/13.9.994 12902399

[B14] Dieterich M. Staab J. P. Brandt T. (2016). Functional (psychogenic) dizziness. *Handb. Clin. Neurol.* 139 447–468. 10.1016/B978-0-12-801772-2.00037-0 27719862

[B15] Edwards M. J. Adams R. A. Brown H. Parees I. Friston K. J. A. (2012). Bayesian account of ‘hysteria’. *Brain* 135 3495–3512. 10.1093/brain/aws129 22641838 PMC3501967

[B16] Edwards M. J. Bhatia K. P. (2012). Functional (psychogenic) movement disorders: merging mind and brain. *Lancet Neurol.* 11 250–260. 10.1016/S1474-4422(11)70310-6 22341033

[B17] Jacob R. G. Woody S. R. Clark D. B. Lilienfeld S. O. Hirsch B. E. Kucera G. D. (1993). Discomfort with space and motion: a possible marker of vestibular dysfunction assessed by the situational characteristics questionnaire. *J. Psychopathol. Behav. Assess.* 15 299–324. 10.1007/bf00965035

[B18] Jáuregui-Renaud K. Sang F. Y. P. Gresty M. A. Green D. A. Bronstein A. M. (2008). Depersonalisation/derealisation symptoms and updating orientation in patients with vestibular disease. *J. Neurol. Neurosurg. Psychiatry* 79 276–283. 10.1136/jnnp.2007.122119 17578858

[B19] Kahneman D. (2003). Maps of bounded rationality: psychology for behavioral economics. *Am. Econ. Rev.* 93 1449–1475. 10.3758/s13423-016-1198-z 27928763 PMC5570804

[B20] Kaski D. Quadir S. Nigmatullina Y. Malhotra P. A. Bronstein A. M. Seemungal B. M. (2016). Temporoparietal encoding of space and time during vestibular-guided orientation. *Brain* 139 392–403. 10.1093/brain/awv370 26719385 PMC4805090

[B21] Kim H. Lee J. Choi J. Kim J. (2020). Etiologic distribution of dizziness and vertigo in a referral-based dizziness clinic in South Korea. *J. Neurol.* 267 2252–2259. 10.1007/s00415-020-09831-2 32300888

[B22] Kinsbourne M. (1977). Hemi-neglect and hemisphere rivalry. *Adv. Neurol.* 18 41–49. 920524

[B23] Nigmatullina Y. Siddiqui S. Khan S. Sander K. Lobo R. Bronstein A. M. (2016). Lateralisation of the vestibular cortex is more pronounced in left-handers. *Brain Stimul.* 9 942–944. 10.1016/j.brs.2016.08.001 27570186

[B24] Rubens A. B. (1985). Caloric stimulation and unilateral visual neglect. *Neurology* 35:1019. 10.1212/wnl.35.7.1019 4010940

[B25] Ruckenstein M. J. Staab J. P. (2009). Chronic subjective dizziness. *Otolaryngol. Clin. North Am.* 42 71–77. 10.1016/j.otc.2008.09.011 19134491

[B26] Sparing R. Thimm M. Hesse M. D. Küst J. Karbe H. Fink G. R. (2009). Bidirectional alterations of interhemispheric parietal balance by non-invasive cortical stimulation. *Brain* 132 3011–3020. 10.1093/brain/awp154 19528092

[B27] Staab J. P. Eckhardt-Henn A. Horii A. Jacob R. Strupp M. Brandt T. (2018). Diagnostic criteria for persistent postural-perceptual dizziness (PPPD): consensus document of the committee for the classification of vestibular disorders of the barany society. *J. Vestib. Res.* 27 191–208. 10.3233/VES-170622 29036855 PMC9249299

[B28] Staibano P. Lelli D. Tse D. (2019). A retrospective. *J. Otolaryngol. Head Neck Surg.* 48 1–8. 30857559 10.1186/s40463-019-0336-9 PMC6413454

[B29] Stone J. Edwards M. (2012). Trick or treat?: showing patients with functional (psychogenic) motor symptoms their physical signs. *Neurology* 79 282–284. 10.1212/wnl.0b013e31825fdf63 22764261

[B30] Ventre-Dominey J. Nighoghossian N. Denise P. (2003). Evidence for interacting cortical control of vestibular function and spatial representation in man. *Neuropsychologia* 41 1884–1898. 10.1016/S0028-3932(03)00126-X 14572522

[B31] Walsh V. (2003). A theory. *Trends Cogn. Sci.* 7 483–488. 14585444 10.1016/j.tics.2003.09.002

[B34] WHO (2015a). *International Classification of Diseases, 11th Edition, Beta Draft, Persistent Postural-perceptual Dizziness*. Available online at: http://id.who.int/icd/entity/2005792829 (accessed September 19, 2015).

[B35] WHO (2015b). *International Classification of Diseases, 11th Edition, Beta Draft, Bodily Distress Disorder*. Available online at: http://id.who.int/icd/entity/767044268 (accessed September 19, 2015).

[B32] Xue H. Chong Y. Jiang Z. D. Liu Z. L. Ding L. Yang S. L. (2018). Etiological analysis on patients with vertigo or dizziness. *Chung-Hua I Hsueh Tsa Chih* 98 1227–1230. 10.3760/cma.j.issn.0376-2491.2018.16.008 29747309

[B33] Zorzi M. Priftis K. Umiltà C. (2002). Brain damage neglect disrupts the mental number line. *Nature* 417 138–139. 10.1038/417138a 12000950

